# A Comparison Of Physiological Responses To Various Intermittent And Continuous Small-Sided Games In Young Soccer Players

**DOI:** 10.2478/v10078-012-0009-5

**Published:** 2012-04-03

**Authors:** Yusuf Köklü

**Affiliations:** 1 Pamukkale University, Schools of Sport Sciences and Technology, Denizli, Turkey.

**Keywords:** Aerobic endurance, training regimens, heart rate, percentage of maximum heart rate, blood lactate

## Abstract

The purpose of this study was to investigate physiological responses to various intermittent and continuous small-sided games (SSGs) – including 2-a-side, 3-a-side, and 4-a-side games – in young soccer players. Twenty soccer players (average age 16.6±0.5 years; mean body height 176.2±4.6 cm; mean body mass 65.9±5.6 kg) voluntarily participated in this study. The subjects underwent anthropometric measurements followed by the YoYo intermittent recovery test. Then, they played intermittent (SSGint) and continuous (SSGcon) 2-a-side, 3-a-side, and 4-a-side soccer-specific SSGs in random order at 2-day intervals. Paired t-tests were used to assess differences between the training regimens (intermittent and continuous) in terms of heart rate (HR), percentage of maximum HR (%HRmax), and blood lactate concentration (LA). The differences in LA, HR and %HRmax between the 2-a-side, 3-a-side and 4-a-side SSGint or the 2-a-side, 3-a-side and 4-a-side SSGcon were identified using a one-way analysis of variance with repeated measures. The results demonstrated that the 3-a-side SSGint and SSGcon measurements were significantly higher than the 2-a-side and 4-a-side games in terms of HR and %HRmax, whereas the 2-a-side SSGint and SSGcon resulted in higher LA responses compared to other SSG types. The study results also demonstrated that SSGint and SSGcon are similar in terms of physiological responses except for 2-a-side game LA responses. The results of this study suggest that both SSGint and SSGcon could be used for the physiological adaptations required for soccer specific aerobic endurance.

## Introduction

Interval training (IT) and continuous training (CT) regimens are used to improve players’ aerobic endurance (Gorostiaga et al., 1991). IT is defined as short bouts of high intensity exercise (80% – 95% VO_2max_) followed by lower intensity rest intervals (Perry at al., 2008). On the contrary to IT, CT is a type of physical effort that involves activity without rest. Therefore, CT is performed over longer durations and at lower exercise intensity (50% – 80% of VO_2max_) than IT (Tuimil et al., 2011).

Traditionally, most coaches used running drills without the ball to develop soccer players’ aerobic endurance (Little and Williams, 2007). However, it is now thought that one can develop technical skills, decision making ability and aerobic endurance in the same training session by using SSGs, which both contribute to the level of physical exertion and ensure more efficient use of the training time available (Little and Williams, 2006; Hill-Haas et al., 2011). Impellizzeri et al. (2006) compared the effects of SSGs versus traditional aerobic running IT on physical fitness and objective measures of match performance in soccer. The results of this study showed that SSGs and traditional aerobic IT make similar contributions to aerobic endurance in young soccer players. Similarly, Hill-Haas et al. (2009a) compared seven weeks of soccer-specific SSG and mixed generic fitness training on selected physiological, perceptual and performance variables. They found that both types of training result in similar levels of improvement in terms of these selected variables.

Intermittent (SSGint) or continuous (SSGcon) type small-sided games are used by coaches in young and senior teams’ soccer training in order to improve aerobic endurance (Fanchini et al., 2011; Koklu et al., 2011; Hill-Haas et al., 2009b; Jones and Drust, 2007). Several studies have previously investigated the effects of SSGint. Fanchini et al. (2011) examined whether an increase in bout duration, using two-, four- and six-minute games, would affect exercise intensity during three bouts of 3-a-side SSGint. Their results showed that an increase in bout duration resulted in a decrease in SSGint intensity especially in the four- and six-minute games. Their results also demonstrated that heart rate during the first bout was significantly lower than in the second and third bouts in 3-a-side SSGint type games. In a different study, Koklu et al. (2011) revealed that HR and %HRmax during the first bout were lower compared to other five bouts in a study comparing 1-a-side, 2-a-side, 3-a-side and 4-a-side SSGint.

There have also been some studies examining physiological responses during SSGcon. Hill-Haas et al. (2009b) examined the acute physiological responses associated with three different SSGcon formats of 2-a-side, 4-aside and 6-a-side games in youth players. Their results showed that, as SSG formats decrease in size and relative pitch area remains constant, overall physiological and perceptual workload increases. Jones and Drust (2007) compared the physiological load, as indicated by heart rate responses, work-rate patterns and technical demands during 4-a-side and 8-a-side SSGcon. Their results indicated that SSGcon activity imposes substantial physiological demands on young players irrespective of the number of players involved in the game.

Although previous studies have investigated physiological responses to either SSGint or SSGcon, only one study has compared the acute physiological responses associated with both SSGcon and SSGint. Hill-Haas et al. (2009c) compared physiological variables between SSGcon and SSGint, focusing on 2-a-side, 4-a-side and 6-a-side games played over the same durations. They found that blood lactate concentration and %HRmax responses were significantly higher in SSGcon than in SSGint. However, no previous studies have compared physiological responses between SSGint and SSGcon for each SSG separately. The purpose of this study was thus to investigate physiological responses to 2-a-side, 3-a-side and 4-a-side SSGint and SSGcon played with different bout durations in young soccer players.

## Material and Methods

### Subjects

Twenty young soccer players (age 16.6±0.5; body height 176.2±4.6 cm; body mass 65.9 ± 5.6 kg; body fat 6.9±1.7 %; HRmax 197.5 ± 6.7 beat x min^−1^) participated in this study voluntarily. All the players had a minimum 5 years of training experience and were the members of the same youth team competing in an elite academy league. This study was approved by the Research Ethics Committee of Pamukkale University, and was consistent with the institutional ethical requirements for human experimentation in accordance with the Declaration of Helsinki. The subjects were fully informed about the procedures to be used and the experimental risk.

### Experimental Approach to the Problem

The 6-week pre-season training period served as a familiarization to all the SSGs and the YoYo intermittent recovery test level 1 (YIRT) for the subjects. At the conclusion of pre-season training, players completed the YIRT and were ranked according to the distance covered. The scores gained on the YIRT were as follows: players who covered the least distance were given a score of 1 and those who covered the most ground were awarded a score of 5. Also, the coach provided an overall subjective technical/tactical skill level of each player using a 5-point scale (from 1 “below average” to 5 “outstanding”). The total score for each payer was the sum of their technical/tactical skill and YIRT scores. In an attempt to avoid skill and fitness mismatches and a consequent imbalance in opposing SSG teams, each side was balanced in terms of the players’ skill and fitness rankings.

The study was conducted over a 2-week period. On the first day, anthropometric measurements (body height, body mass, skinfold thickness, circumference measurements) were taken for each player. They were followed by the YIRT.

Then, intermittent and continuous 2-a-side, 3-aside, and 4-a-side soccer-specific SSGs were organized in random order with 2-day intervals. Each SSG was played after a 20-minute warm-up, which consisted of low intensity running, striding, and stretching. The HRmax for each player was determined during the YIRT; LA concentrations were taken after the SSGs and HRs were measured during the SSGs. The YIRT and SSGs were carried out on a synthetic grass pitch between 4 and 6 pm.

### Procedures

#### YoYo Intermittent Recovery Test

The YIRT consists of repeated 20-m runs back and forth between the starting, turning, and finishing lines, and at a progressively increased speed, which is controlled by audio beeps from a tape recorder. The test was performed on a synthetic grass field in groups of 6 players, as suggested by Bangsbo et al. (2008). The HR was measured and stored using Polar S810 HR monitors (Polar Electro OY, Kempele, Finland) throughout the test. Stored data were transferred to the computer and filtered by Polar Precision Performance SoftwareTM (PPP4, Finland). The highest HR measurement was recorded as YoYo HRmax.

#### Small-Sided Games

Table 1 shows the number of bouts, bout duration (min), total time (min), pitch dimension (length x width), and relative pitch size (m^2^) for the SSGint and SSGcon. The soccer players were asked to perform at maximum effort during the games. No specific rules were utilized in the games to influence their intensity, but extra balls were placed in the goals and along the side lines surrounding the entire pitch to ensure that there were no breaks in play. Moreover, the coaches constantly encouraged the players verbally during the games. SSGcon and SSGint were played in random order at two day intervals without a goalkeeper.

#### Heart Rate Monitoring

Heart rate was recorded at 5-s intervals during each small-sided game via short-range radio telemetry (Polar Team Sport System, Polar Electro Oy, Finland). The heart rate monitors were also worn during the YIRT to determine each player’s maximum heart rate (HRmax). The HR was stored by Polar S810 HR monitors throughout the games and transferred to the computer and filtered by Polar Precision Performance Software TM (PPP4, Finland). The mean HR for each SSGint was calculated by taking the means of the 3 bouts played (HRgame). The mean HR for each SSGcon was calculated by taking the means of all games (HRgame). The %HRmax was then calculated by formula 1 for each SSGs.

%HRmax : (HRgame / YoYo HRmax) × 100 [Formula 1]

#### Blood Sampling

Blood lactate samples were taken within two minute of the end of each SSG. The twenty-five microliter samples, taken from the ear lobes, were kept in YSI Preservative Kits (Code 2315, YSI Incorporated Life Sciences, Yellow Springs, OH, USA) during the games. Once the games were finished, lactate analyses were carried out using an YSI 1500 SPORT analyzer (YSI Incorporated Life Sciences). The calibration of the analyzer was carried out according to the manufacturer’s instructions.

#### Statistical Analysis

The data are reported as means and standard deviations. Before using parametric tests, the assumption of normality was verified using the Shapiro-Wilk test. A paired t-test was performed on each dependent variable, including HR, %HRmax and LA differences between the training methods (intermittent and continuous). A one-way repeated-measures analysis of variance was performed on each dependent variable, including HR, %HRmax and LA either between the 2-a-side, 3-a-side and 4-a-side SSGint or bewteen the 2-a-side, 3-a-side and 4-a-side SSGcon. The Bonferroni Post Hoc test was applied to make a pairwise comparison between the different levels of within subjects’ factors (games). The level of statistical significance was set at p < 0.05.

## Results

Table 2 shows average HR, %HRmax, and LA responses of the players to SSGint for 2-a-side, 3-a-side, and 4-a-side games. There were significant differences between 2-a-side, 3-a-side, and 4-a-side SSGint in terms of HR (F=11.376; p=0.001; η2 = 0.374), %HRmax (F=11.593; p=0.001; η2 = 0.379) and LA (F=4.193; p=0.023; η2 = 0.181).

Table 3 shows average HR, %HRmax, and LA responses of the players to SSGcon for 2-a-side, 3-a-side, and 4-a-side games. There were also significant differences between 2-a-side, 3-a-side, and 4-a-side SSGcon in terms of HR (F=8.501; p=0.001; η2 = 0.309), %HRmax (F=8.452; p=0.001; η2 = 0.308) and LA (F=3.342; p=0.046; η2 = 0.150).

**Figure 1:**
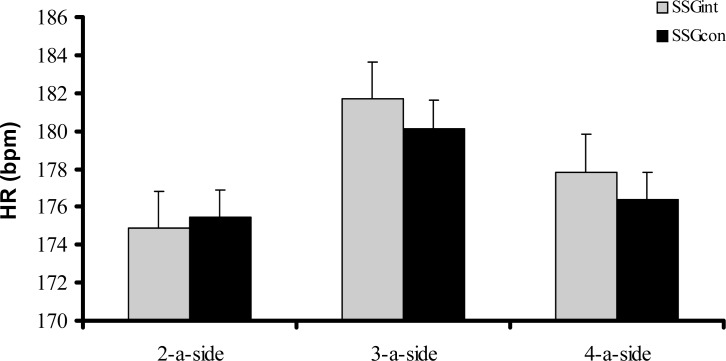
Heart rate (HR) of soccer players after intermittent (SSGint) and continuous (SSGcon) games played in 2-a-side, 3-a-side, and 4-a-side formats

No significant differences were found between SSGint and SSGcon for 2-a-side (t=−0.311; p= 0.760), 3-a-side (t=1.447; p= 0.164), and 4-a-side (t=1.990; p= 0.061) formats in terms of HR (Figure 1).

**Figure 2:**
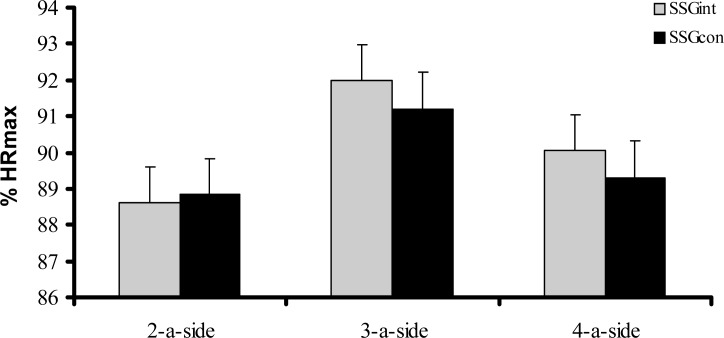
Percentage of maximum heart rate (%HRmax) in soccer players after intermittent (SSGint) and continuous (SSGcon) 2-a-side, 3-a-side, and 4-a-side games

There were no significant differences between SSGint and SSGcon including 2-a-side (t=−0.242; p= 0.811), 3-a-side (t=1.465; p= 0.159), and 4-a-side (t=1.962; p= 0.065) in terms of %HRmax (Figure 2).

**Figure 3:**
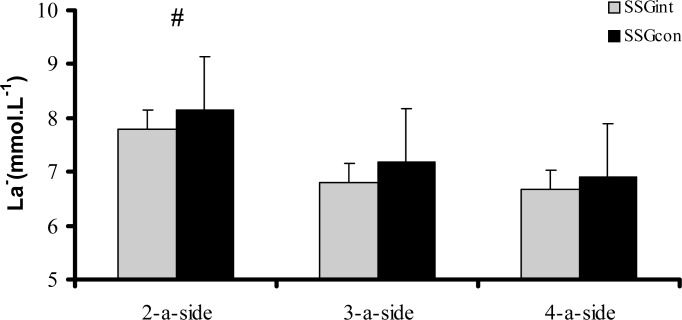
Blood Lactate (LA) concentrations in soccer players after intermittent and continuous 2-a-side, 3-a-side, and 4-a-side games. # p=0.027

Finally, there were significant differences between SSGint and SSGcon for the 2-a-side format (t=−2.401; p= 0.027) in terms of LA concentration, whereas no significant differences were found between SSGint and SSGcon for 3-a-side (t=−1.931; p= 0.069) and 4-a-side (t=−1.207; p= 0.242) games (Figure 3).

## Discussion

The aim of this study was to investigate physiological responses in young soccer players to various SSGint and SSGcon played with different bout durations. The study results demonstrate that the 3-a-side SSGint and SSGcon responses were significantly higher than the 2-a-side and 4-a-side games in terms of HR and %HRmax, whereas the 2-a-side SSGint and SSGcon resulted in higher LA responses compared to the other SSG formats. The present study findings also indicate that SSGint and SSGcon are similar in terms of HR, %HRmax and LA responses except for the 2-a-side game LA responses.

High intensity aerobic intervals at 90–95% HRmax are an effective tool for increasing aerobic endurance in soccer players (McMillan et al., 2005). Although it is known that SSGint training leads to improvements in both aerobic endurance and match performance (Jones and Drust, 2007; Kelly and Drust, 2009), too few studies have focused on SSGcon. Only Hill-Haas et al.’s (2009c) study compared acute physiological responses associated with both SSGcon and SSGint. They found that SSGcon training elicited a significantly higher %HRmax response than SSGint. However, this study indicated that there was no significant difference between SSGcon and SSGint in terms of HR and %HRmax responses. This difference in findings is most likely due to differences in terms of number of bouts and bout duration. The present study also found 3-a-side SSGint and SSGcon HR and %HRmax responses to be significantly higher than those in 2-a-side and 4-aside formats. One of the reasons for this finding could be that 3-a-side SSGint and SSGcon have a lower relative pitch size than 2-a-side and 4-a-side SSGint and SSGcon. When the pitch size per player is increased, the intensity and the involvement in the game might be decreased. In line with the present study, Jones and Drust (2007) found similar HR responses during the 4-a-side and 8-a-side SSGcon of ten-minute duration. However, Hill-Haas et al. (2009b) found that, as SSG formats decrease in size and relative pitch area remains constant, %HRmax responses increase for SSGcon of twenty-four minute duration. Previous studies on SSGint have found similar HR and %HRmax responses to those in the current study (Hill-Haas et al., 2010; Koklu et al., 2011).

The present study has demonstrated that lactate responses in SSGcon are higher than in SSGint. Two minutes of recovery between each bout in SSGint may help to remove the lactate and reduce its production, whereas the lack of a rest period in SSGcon may result in the accumulation of lactate. This finding is similar to the results of the study by Hill-Haas et al. (2009c). They revealed that, although the difference between SSGcon and SSGint LA responses was not significant, SSGcon did show higher LA concentrations. In a separate study, Hill-Haas et al. (2009b) found that as SSG formats decrease in size and relative pitch area remains constant, LA responses increases during SSGcon of twenty-four-minute duration. The present study also showed that decreasing the playing area and reducing the number of players leads to an increase in LA concentration for both SSGcon and SSGint. Similarly, Koklu et al. (2011) found that decreasing the number of players resulted in increased LA production during SSGint. According to these results, 2-a-side SSGint and SSGcon are more anaerobic than 3-a-side and 4-a-side SSGint and SSGcon. Technical actions such as the number of ball contacts and the number of tackles may increase LA concentration, especially in SSGs including fewer players. No other studies have reported LA responses associated with SSGcon.

## Conclusion

SSGs studies have generally examined interval formats. However, the present study has shown that both SSGint and SSGcon could be used for the physiological adaptations required for soccer specific aerobic endurance. Our findings indicate that 3-a-side and 4-a-side SSGint and 3-a-side SSGcon could be used to improve maximum oxygen uptake (> 90% of HRmax ), whereas 4-a-side SSGcon games might be used to develop the anaerobic threshold (85–90% of HRmax). In addition, 2-a-side SSGint and SSGcon games can be used to improve lactate tolerance. Unfortunately, we were not able to measure the distance covered at various running speeds and technical actions, and therefore future studies which investigate time-motion analysis and technical actions during the SSGcon and SSGint are needed.

## Figures and Tables

**Table 1:** Established characteristics of intermittent and continuous small-sided games

	2-a-side		3-a-side		4-a-side	
	Intermittent	Continuous	Intermittent	Continuous	Intermittent	Continuous
Number of Bouts	3	No bout	3	No bout	3	No bout
Bout Duration (min)	2	6	3	9	4	12
Total Time (min)	6		9		12	
Pitch Dimension (length x width) (m x m)	15 × 20		18 × 24		24 × 36	
Relative Pitch Size (m^2^)	1:75		1:72		1:108	
Coach Encouragement	Yes		Yes		Yes	
Goalkeeper	No		No		No	

**Table 2:** Soccer players’ average HR, %HRmax, and LA responses SSGint *

	HR (b·min^−1^)	%HRmax	LA ( mmol·L^−1^)
2 -a-side	174.9±5.4	88.6±3.8	7.8±1.6
3 -a-side	181.7±5.7¥	92.0±2.0¥	6.8 ±1.3#
4 -a-side	177.8±5.9	90.1±2.5	6.7±1.5

* Values are given as mean ± SD; HR: Heart rate; %HRmax: Percentage of maximum heart rate; LA : Blood lactate; SSGint : Intermittent small sided games;

¥ Significant difference from 2 -a-side and 4 -a-side, p=0.001;

# Significant difference from 2 -a-side, p=0.023

**Table 3:** Soccer players’ average HR, %HRmax, and LA responses SSGcon^*^

	HR (b·min^−1^)	%HRmax	LA ( mmol· L^−1^)
2 -a-side	175.4±7.7	88.8±3.2	8.1±1.7
3 -a-side	180.1±6.7^†^	91.2±2.6^†^	7.2±1.5#
4 -a-side	176.3±5.3	89.3±2.7	6.9±1.8

* Values are given as mean ± SD; HR: Heart rate; %HRmax: Percentage of maximum heart rate; LA : Blood lactate; SSGcon : Continuous small sided games;

† Significant difference from 2 -a-side and 4 -a-side, p=0.001;

# Significant difference from 2 -a-side, p=0.046
